# Mitigating anthropogenic climate change with aqueous green energy

**DOI:** 10.1038/s41598-025-86042-7

**Published:** 2025-01-11

**Authors:** Sophia T. Olim, Anna Nickoloff, Leslie J. Moffat, Andrew J. Weaver, Michael Eby

**Affiliations:** School of Earth and Ocean Sciences, University of Victoria, PO Box 1700, Victoria, BC V8W 2Y2 Canada

**Keywords:** Climate change, Climate and Earth system modelling, Physical oceanography

## Abstract

Reaching net zero emissions and limiting global warming to 2 °C requires the widespread introduction of technology-based solutions to draw down existing atmospheric levels and future emissions of CO_2_. One such approach is direct air CO_2_ capture and storage (DACCS), a readily available, yet energy-intensive process. The combination of DACCS and ocean thermal energy conversion (OTEC) allows for independently powered carbon capture plants to inject concentrated carbon into deep marine sediments where storage is generally safe and permanent. OTEC is a form of electricity production that exploits the temperature difference between deep and shallow ocean waters, and can power DACCS on floating platforms at a price competitive with coal-generated electricity. Here we highlight the scale of the challenge facing society. We show that a safe and sustainable level of OTEC-generated electricity powering DACCS for 70 years could result in up to a 35% decrease in the relative global mean temperature warming compared to a business-as-usual emissions scenario.

## Introduction

The 2018 Intergovernmental Panel on Climate Change report noted the need for widespread negative emission technologies in order to meet the goal of the 2015 Paris Accord and limit warming to 1.5 °C since preindustrial times^1^. This is largely because natural greenhouse gas sinks cannot keep up with the rate of anthropogenic greenhouse gas emissions. In direct air CO_2_ capture and storage (DACCS) atmospheric CO_2_ is captured through a series of chemical processes using liquid solvents or solid sorbents^2^ and subsequently liquidized. Following compression into a supercritical liquid, the CO_2_ can be injected into suitable intact marine sediments where storage is generally safe and permanent^3^. Ocean floor sedimentary basins along passive continental shelves, such as depleted or semi-depleted oil and gas fields, are the most geologically sound choice for this^4^. In these basins, the liquidized CO_2_ is injected into available pore space below an impermeable capstone. Depleted oil and gas fields make ideal sites for long-term CO_2_ storage as geologic data suggest these areas have good storage and trapping characteristics^4,5^.

Ocean thermal energy conversion (OTEC) is a form of marine renewable energy that uses the natural thermal gradient between cold deep ocean water and warm surface ocean water to power a heat engine^6^. It was conceived in 1881 by Jacques-Arsène D’Arsonval, who recognized the net power depended on the magnitude of the temperature difference between the warm and cold water. To maximize its potential, he proposed warm water be drawn from the surface near the equator and cold water from the same latitude but at great depths^7^. In 1930, D’Arsonval’s student, Georges Claude, set this idea into practice in Cuba by building the first pilot plant.

OTEC consists of pumping warm surface water from the mixed layer and using its heat to evaporate a working fluid inside a heat exchanger. The working fluid in vapour form then passes through a turbine where it expands, converting thermal energy into kinetic energy and then electrical energy by way of a generator. The working fluid then proceeds to a second heat exchanger where it is cooled by cold deep seawater, pumped in from approximately 1000 m deep, and condensed back into liquid form before restarting the cycle. After the warm and cold water have passed through their respective heat exchangers, the two are mixed and discharged at the level of neutral buoyancy. While the use of a non-seawater working fluid improves the process’ efficiency, it can also be conducted using seawater, producing freshwater as a by-product.

While initial and ongoing studies consider OTEC as a method of clean electricity generation^8,9^, more recently OTEC has also been suggested as a clean energy source for CO_2_ negative emissions technology^6^. OTEC generated electricity could be used to power liquid solvent based DACCS in terrestrial facilities or solid sorbent based DACCS in marine conditions. The captured CO_2_ could then be transported and sequestered in offshore storage sites.

The levelized cost of electricity (LCOE) of OTEC is competitive with that of coal-fired electricity production. The inflation adjusted (using the U.S. Bureau of Labor Statistics online CPI inflation calculator) LCOE of OTEC ranges from 0.04 to 0.24 USD_2023_/kWh^10–12^ prior to considering any discounts or subsidization. The 2020 LCOE of coal-generated electricity in the US is between 0.075 and 0.116 USD_2020_/kWh (Table 3.12 in ^13^) assuming a 3% discount rate, or equivalently and adjusted for inflation, between 0.09 and 0.14 USD_2023_/kWh.

Approximations of how much power OTEC is able to produce per year without significant environmental impact vary greatly. The discrepancy comes mainly from the large amount of uncertainty surrounding the limiting factor for reservoir renewal. Other concerns include deep water formation^14^, environmentally safe levels of OTEC production density^15^, and possible disruptions of the oceanic thermohaline circulation^6,16^.

Consistent with^17^, the sensitivity analysis we performed using the UVic Earth System Climate Model (UVic ESCM) revealed that 3 TW of OTEC electricity production a year could be sustained for at least 500 years without substantial adverse effects on the ocean circulation or its physical and chemical properties. The combination therefore of DACCS and OTEC offers an exciting new approach for independently powered carbon capture plants to inject concentrated supercritical CO_2_ into the deep ocean in an economically and environmentally beneficial approach.

In this report, we use the UVic ESCM to explore the feasibility of fueling negative emission technology with aqueous clean energy, in order to significantly decrease the global temperature increase that would otherwise occur.

## Methodology

The UVic ESCM consists of an energy-moisture balance atmospheric model, a dynamic-thermodynamic sea-ice model^18–20^, and a primitive equation oceanic general circulation model^21^. The UVic ESCM has fully coupled representations of the oceanic and terrestrial carbon cycles and global coverage with a horizontal resolution of 3.6 degrees (zonal) by 1.8 degrees (meridional) (see ref. ^22^ for details). The model was initialized with a 10,000-year spin-up at the year 850. From 850 to the year 2005, historical forcing (land cover change, solar, volcanic, aerosol, CO_2_ and other GHGs) was used to force the model. In addition, over the period 2005 to 2100 changing CO_2_, land surface, aerosol and other greenhouse gas forcing followed the Intergovernmental Panel on Climate Change Representative Concentration Pathway 8.5 (IPCC RCP 8.5; ^23^). From 2000 to 2100, the equivalent CO_2_ emissions were diagnosed^24^ and then used in the subsequent sensitivity experiments. This allowed the model to react to the sequestration of CO_2_ rather than be constrained by specific concentrations.

To determine useful OTEC plant locations, the vertical temperature gradient in each viable ocean grid cell was calculated and OTEC plants were placed first in locations with the greatest temperature gradient. This is because OTEC is most efficient in areas with large temperature gradients. OTEC requires a thermal gradient of at least 18 to 20 °C between where surface water uptake occurs and where the deep-water uptake occurs^17,25^. If the temperature gradient fell below 18 °C or the power generated by each OTEC plant fell below 75 MW the plant in our model was “decommissioned” and relocated. Each OTEC plant was assumed to require a minimum area of 50 km^2^. The net power generated by each deployed OTEC plant was determined as follows (see ref. ^26^ for more details):

$$\:{P}_{net}=\:{\omega\:}_{cw}\rho\:{c}_{p}{\epsilon\:}_{tg}\left(\frac{9}{80}\frac{{\left({\Delta\:}T\right)}^{2}}{T}-\frac{9}{200}\right)$$

where $\:{\omega\:}_{cw}$ is the volume flow rate of OTEC deep seawater, $\:\rho\:$ is the mean seawater density (1,025 kg m^− 3^), $\:{c}_{p}$ is the specific heat (4000 J kg^− 1^ K^− 1^), $\:{\epsilon\:}_{tg}$ is the turbo-generator efficiency (0.75), $\:T$ is the intake seawater temperature, and $\:{\Delta\:}T$ is the temperature difference between surface and deep seawater intakes.

The calculated emission reduction was incorporated by factoring it into transient CO_2_ atmospheric forcing where all available OTEC-generated electricity was used to power DACCS. Two approaches were taken. First, we used estimations of energy required for DACCS from the Squamish Carbon Engineering plant, where 366 kWh of electricity and 5.6 GJ of natural gas are needed to sequester one tonne of CO_2_ (see ref. ^27^ for more details). Here we note that the Carbon Engineering plant uses a liquid solvent system and so natural gas combustion is required to create the very high temperatures needed. While we recognize technological barriers exist in creating such high temperatures from renewable energy, research and development in overcoming these barriers is advancing^28^. In the two experiments discussed below, we assume in one case (*ODLMit* — *O*TEC/*D*ACCS *L*ow *Mit*igation) that all energy is produced from OTEC (1921 kWh per tonne of CO_2_ sequestered). In the other case (*ODHMit* — *O*TEC/*D*ACCS *H*igh *Mit*igation), we assume that local reserves of natural gas are available so OTEC is solely used to provide the required electricity (366 kWh per tonne of CO_2_ sequestered). In this case the emissions from natural gas combustion are also sequestered.

**Fig. 1:**
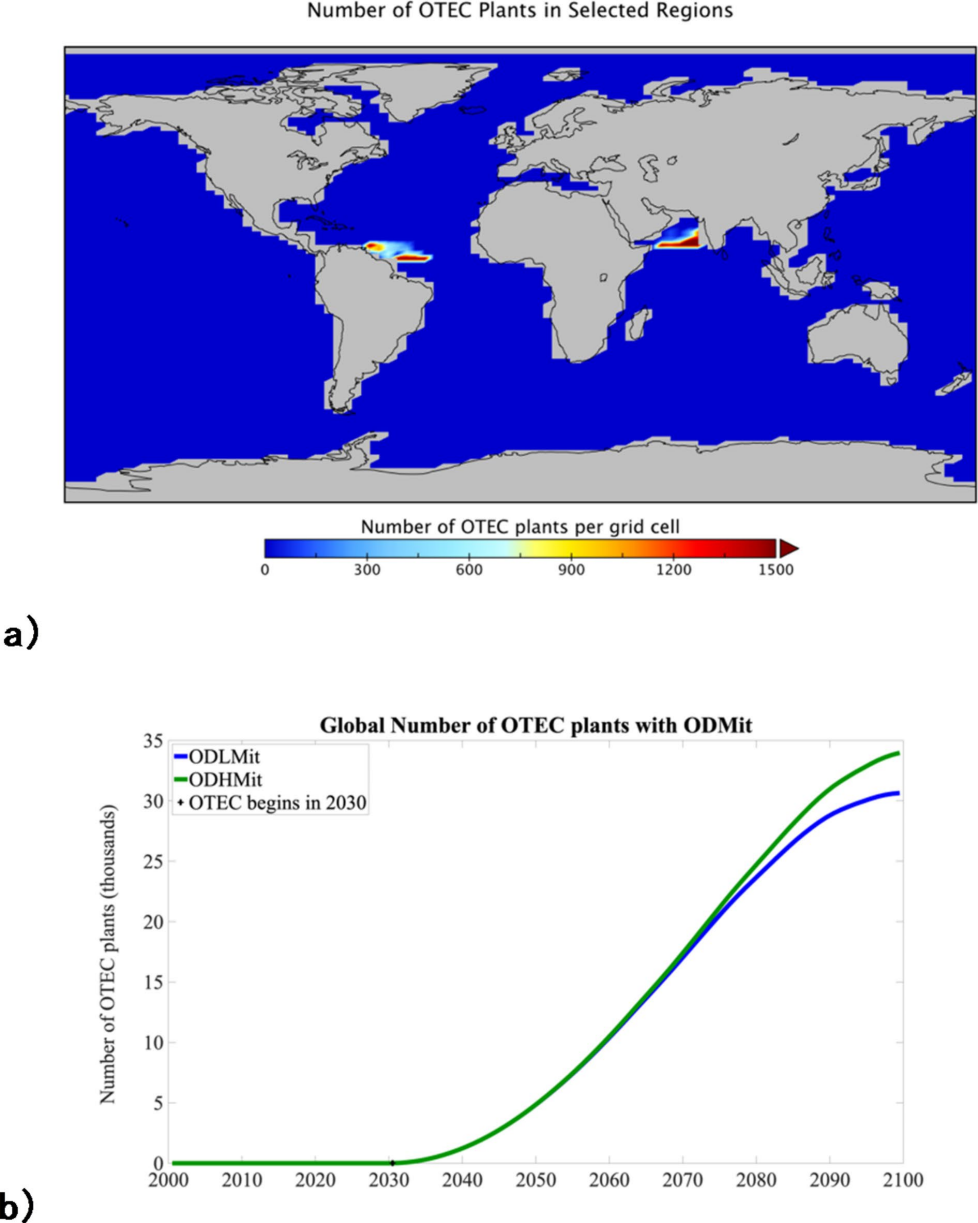
(**a**) Map of the selected geologic regions with the number of OTEC plants per grid cell in 2100 for the *ODLMit* experiment. (**b**) Number of OTEC stations in the model as a function of time from 2000 to 2100. The total number of OTEC stations is lower in *ODLMit* than *ODHMit* as the greater surface water temperatures in the scenario allow plants to function more efficiently. Figure produced using Panoply, a freely available NASA GISS data viewer: https://www.giss.nasa.gov/tools/panoply/.

Two deep ocean CO_2_ storage sites were selected: (1) off the east coast of Oman in the Arabian Sea; (2) off the northern coast of Venezuela in the western equatorial Atlantic (Fig. 1). Both sites are excellent target basins for long-term CO_2_ storage because of their stability and structural integrity^29^. This is in large part due to their location on the passive edge of stable continental plates. This and their location within the suitable latitude range for OTEC is why these sites were selected. While there is a need to obtain more information on the storage capacity of these selected areas, their known large amounts of oil and gas reserves suggest that there would be the capacity to store large amounts of CO_2_.

In this report, three model integrations are compared. All experiments use the diagnosed emissions from the 21st century integration driven by RCP 8.5. The IPCC RCPs were used extensively in models run for the IPCC AR5 and include a time series of emissions and GHG concentrations from 2005 to 2100 with extensions to 2300^1^. RCP 8.5 represents a business-as-usual scenario where there are high emissions without effective climate change mitigation practices. In this scenario, the radiative forcing reaches 8.5 W/m^2^ by 2100^1^.

## Results

**Fig. 2:**
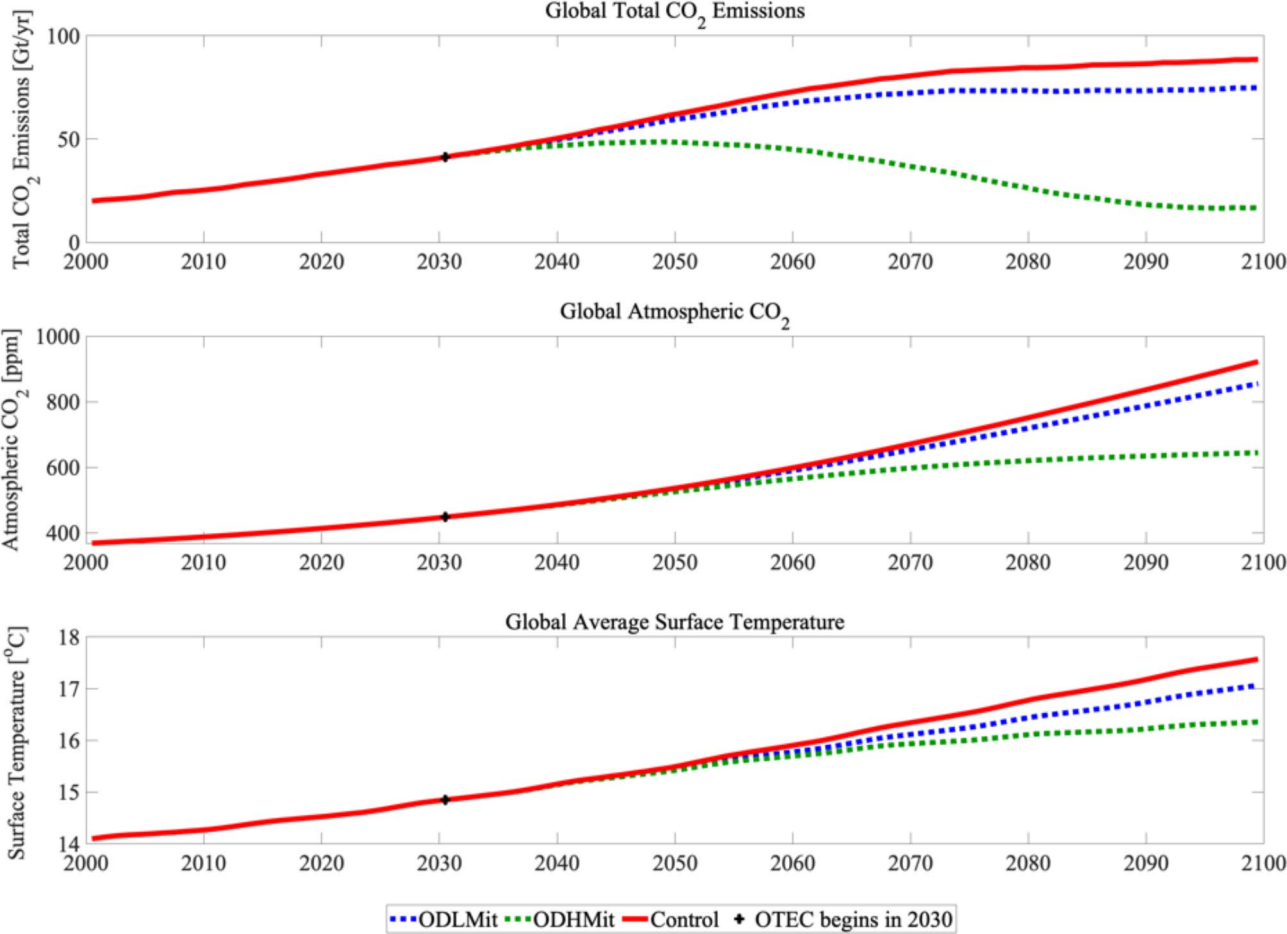
Results from the *Control* (red solid line), *ODHMit* (green dotted line) and *ODLMit* (blue dotted line) experiments over the 21st century. (**a**) CO_2_ emissions in GtCO_2_/yr; (**b**) atmospheric CO_2_ concentration in ppm; (**c**) globally-averaged surface temperature in °C. OTEC deployment begins in 2030 and ends up producing 3TW of power for DACCS by 2100.

To explore the climate effects of widespread OTEC deployment coupled with DACCS, we first conducted a *Control* experiment where the model was integrated from 2000 to 2100 using the diagnosed emissions from the RCP 8.5 spin-up (Fig. 2a). In this *Control* experiment the atmospheric CO_2_ concentration increases to 922 ppm in 2100, 553 ppm higher than in year 2000. Concomitant with the increase in greenhouse gas forcing, the global mean surface air temperature increased by 3.5 °C from 14.1 °C in year 2000 to 17.6 °C in year 2100 (Fig. 2b). In *ODLMit*, OTEC power production was initiated in year 2030 and grew following a raised negative cosine function until the number of plants deployed (~ 30,700 plants producing on average 98 MW each) at year 2100 met the requested power demand of 3 TW (see Fig. 1b). All OTEC power is directed towards DACCS and in year 2100, at peak OTEC production, 14 GtCO_2_ is being sequestered annually (Fig. 2a).

*ODHMit* follows the same experimental design as *ODLMit* with the difference being that OTEC energy is used to only power the required 366kwh of electrical energy per tonne of captured CO_2_. In this case, ~ 33,000 plants, each producing on average 90 MW, met the requested 3TW power demand at 2100 (see Fig. 1b). In 2100, 72 GtCO_2_ is being sequestered annually in *ODHMit* (Fig. 2a). This is nearly twice the anthropogenic CO_2_ emissions in year 2030 diagnosed from the RCP 8.5 spin-up (41 GtCO_2_) and twice present-day anthropogenic emissions of CO_2_ (37 GtCO_2_). In total, 471 GtCO_2_ and 2,446 GtCO_2_ were sequestered from 2030 to 2100 in *ODLMit* and *ODHMit*, respectively. These are well within estimates of available capacity for CO_2_ storage in deep saline formations and available oil and gas fields^29^.

**Fig. 3:**
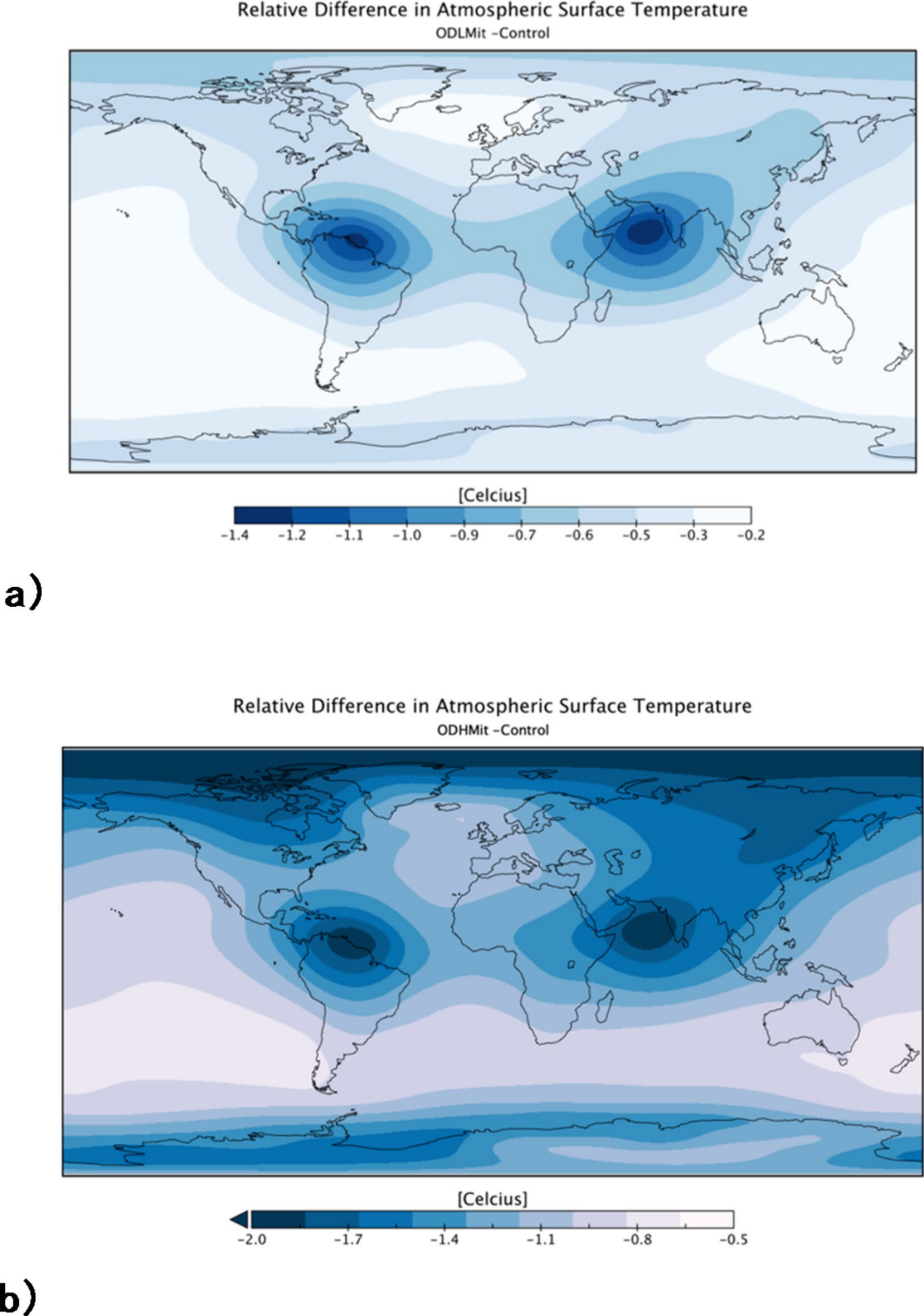
Difference in surface air temperature (°C) at year 2100 between (**a**) the *ODLMit* and *Control* experiments and (**b**) the *ODHMit* and *Control* experiments. OTEC deployment begins in 2030 and ends up producing 3TW of power for DACCS by 2100. Figure produced using Panoply, a freely available NSAS GISS data viewer: https://www.giss.nasa.gov/tools/panoply/.

In the *ODHMit* experiment, global CO_2_ emissions continue to rise from 2030 to 2050, before beginning to decline. This occurs because OTEC power production, and therefore the amount of DACCS that is powered, increases with time. By 2100, net CO_2_ emissions in the *ODHMit* experiment are reduced to only 17 GtCO_2_/year (Fig. 2a) and the resulting atmospheric CO_2_ concentration is 645 ppm (Fig. 2b). Compared to the results from *Control*, this represents a 278 ppm reduction in atmospheric CO_2_ and a 72 Gt reduction in annual anthropogenic CO_2_ emissions. In the case of *ODLMit*, where the majority of OTEC energy is used to replace the natural gas energy component in *ODHMit*, net CO_2_ emissions did not drop to the same extent. In *ODLMit*, CO_2_ emissions only reduced to 74.8 GtCO_2_/year and the atmospheric CO_2_ concentration reached 855.6 ppm at year 2100. Compared to the results from *Control*, this represents a 66.8 ppm reduction in atmospheric CO_2_ and a 13.6 Gt reduction in annual anthropogenic CO_2_ emissions.

The growing reduction in net 21st -century anthropogenic CO_2_ emissions after the deployment of OTEC and DACCS leads to a concomitant increasing reduction in atmospheric surface temperature warming (Fig. 2c) compared to the case with no CO_2_ sequestration. The resulting 2100 globally-averaged atmospheric surface air temperature in *ODLMit (ODHMit)* is 17.1 °C (16.4 ºC), a 0.5 °C (1.2 °C) reduction from *Control*, the experiment without any CO_2_ sequestration (Figs. 2c and 3). Nevertheless, global mean surface air temperatures still rose 2.3 °C in *ODLMit* and 1.5 °C in *ODHMit* more than the 2030 average (14.8 °C), although the rise is significantly lower than in *Control* where the increase above 2030 was 2.7 °C. This relative temperature decrease is felt across the globe but is concentrated in the areas where OTEC is operational and over the Arctic, where climate feedbacks are strongest (Fig. 3).

## Discussion

Based on the potential for mitigating anthropogenic climate change suggested by our analysis, we chose to perform initial calculations estimating the economic feasibility of implementing OTEC coupled with DACCS at the suggested scale. While we recognize that such estimates are highly uncertain and far too general to be definitive, emerging literature can help guide us. Below we first explore the cost of deploying OTEC, followed by the cost of installing DACCS in association with OTEC.

### The cost of OTEC

The cost to build OTEC plants varies greatly depending on the location, scale, and technology used to build these plants. At this time there is limited quantitative cost data available for OTEC, with most cost references based on feasibility studies from limited sources. Ref.^10^ attempted to provide an overview of the available cost projections for a range of OTEC plants, spanning 5 to 250 MW capacity, based on data from^12^. They found that due to the large overhead cost, small-scale OTEC plants (those capable of producing less than 10 MW power) have comparatively higher capital costs per kW than larger (10 to 100 MW) OTEC plants. They suggested that the capital cost of an OTEC plant with 100 MW capacity would range from USD_2010_ 5,000/kW to 15,000/kW. These capital costs include plant components such as pumps, heat exchangers, and components for thermodynamic cycles which are considered predictable costs since these components are commercially available for other offshore operations. Other costs, such as labour, were estimated based on USD_2010_ prices. Therefore, for an OTEC plant with 90 MW capacity, the capital cost per plant would be USD_2010_ 500 million to 1.5 billion^10^. Using inflation-adjusted conversion rates from the U.S. Bureau of Labor Statistics, this is equivalent to 700 million to 2.1 billion USD_2023_. To produce 3 TW of electricity in *ODHMit* using 90 MW OTEC plants, we needed 33,000 plants, whereas 30,700 plants producing on average 98 MW, were needed in *ODLMit*.

Using the lower and the upper bound of these estimates (500 million USD_2023_ and 2.1 billion USD_2023_, respectively), the required 33,000 OTEC plants in *ODHMit* would cost between 17 trillion USD_2023_ and 69 trillion USD_2023_, respectively. The 30,700 plants in *ODLMit* would cost between 15 trillion USD_2023_ and 64 trillion USD_2023_. These numbers do not consider the decrease in price that would occur as the OTEC plants become more standardized (economy of scale) or the possibility of attaching OTEC plants to decommissioned oil and gas platforms. Ref.^10^ estimated that closed-cycle OTEC plants (where ammonia with a low boiling point is used as the working fluid) are less expensive than open-cycle (where seawater is used as the working fluid) designs. However, open-cycle OTEC plants produce fresh water as a by-product whereas closed-cycle plants do not, so the economic benefits of freshwater production may make open-cycle OTEC systems more economically viable in the long term, particularly for plants located near small island nations or the coast.

Once OTEC is operational, the LCOE also depends on interest rates and subsidies. For a ~ 100 MW plant, it has been estimated that the LCOE could range from 0.09 to 0.24 USD_2023_/kWh^10–12^. Additionally^11^, estimated that obtaining financing from government bonds (with lower interest rates than commercial loans) could reduce the LCOE of OTEC power generation to around 0.04 USD_2023_/kWh for a ~ 100 MW OTEC plant. For comparison, ref.^13^ recently estimated the LCOE for US-based coal-powered electricity as 0.09 to 0.14 USD_2023_/kWh. Therefore, the LCOE of OTEC-generated electricity could be considered competitive with the LCOE of coal-generated electricity.

### The cost of DACCS

One of the major factors in determining the cost of DACCS is the cost of electricity and thermal energy used to power the system. Since the energy requirement for CO_2_ capture and storage can be significantly larger than for other emission control systems, how DACCS is powered is an important consideration to ensure the process is both economically profitable and environmentally beneficial. Due to the number of factors influencing the cost of DACCS, there is little consensus on estimates of the technology’s cost. Cost estimates based on simple scaling relationships, yield results from ~ 50 to ~ 1,200 USD_2023_ per tonne CO_2_ sequestered^27,30–34^.

In April 2022 the International Energy Agency published a report^28^ exploring the role that direct air capture could play in assisting the world reach net zero emission targets. They noted that only 18 such plants existed at the time of publication, although governments and industry had committed billions in direct air capture technology investment in the years ahead. While the cost per tonne of CO_2_ removed is presently high, the IEA estimated that for a large-scale plant built in 2022, the cost would be between 125 and 335 USD per tonne CO_2_ captured. They further argued that by 2030, costs for DAC could fall to below $100 per ton of CO_2_ capture.

Carbon Engineering, based in British Columbia in Canada suggested that direct air capture on land could be achieved for 94 to 232 USD_2018_ per tonne (equivalent to 114 to 280 USD_2023_; ^27^). This is much lower than the previous American Physical Society estimate^35^ of 600 USD_2011_ per tonne CO_2_ (equivalent to 830 USD_2023_), suggesting, and consistent with ref.^28^, that the price of direct air CO_2_ capture is rapidly decreasing with technological advances and will continue to do so.

If we use the range of land-based direct air capture costs from the IEA report (between 125 USD_2023_ and 335 USD_2023_ per ton of CO_2_ captured, respectively), the cost to extract the 2,446 Gt of CO_2_ in *ODHMit* through direct air capture converts to between 305 trillion and 819 trillion USD_2023_. In the case of *ODLMit*, the cost to extract the 471 GtCO_2_ would be between 59 trillion and 158 trillion USD_2023_. Considering the decrease in price that would occur with electricity coming from OTEC rather than the electrical grid, these costs would decrease further. We recognize that the above estimates are for land-based direct air capture and that these costs could be expected to be different in the marine environment.

The technology for injecting CO_2_ into depleted or nearly depleted oil and gas reservoirs is mature and has been successfully used for decades for enhanced oil recovery. This method of carbon injection occurs when liquidized CO_2_ is injected into the oil-bearing formation to lower the viscosity of the oil, therefore allowing the oil to flow more easily to the oil well^36^. The 2019 cost for land-based enhanced oil recovery CO_2_ injection was estimated to be about 40 USD_2019_ per tonne of CO_2_ stored^37,38^, although the IEA report^28^ notes that in the US, land-based storage can be achieved for less than 10 USD while offshore storage cost below 35 USD per ton of CO_2_ stored. Assuming 35 USD_2023_ per ton of CO_2_ stored, the total cost to store 2,446 GtCO_2_ is 86 trillion USD_2023_ and to store 471 GtCO_2_ is 16 trillion USD_2023_.

### Total cost

The capture and compression/liquefaction of CO_2_ are expected to be the dominant costs involved in DACCS, followed by transport costs^39^. With OTEC powering DACCS directly above tropical depleted to semi-depleted oil and gas fields, the cost associated with the transport of the liquified CO_2_ would be eliminated. We can also assume, due to the presence of pre-existing technology, that the cost of CO_2_ injection into marine sites is similar to that of DACCS on land and so use estimates from existing enhanced oil recovery operations. Taken together, we estimate the total cost for 33,000 90 MW OTEC plants that sequester 2,446 Gt (*ODHMit*) of CO_2_ over a 70-year period (Figs. 1b and 2a) using existing enhanced oil recovery technology ranges from 407 trillion to 973 trillion USD_2023_ (equivalent to between 166 and 398 USD_2023_ per ton of CO_2_ sequestered). The total cost for 30,700 98 MW OTEC plants that sequester 417 GtCO_2_ (*ODLMit*) over a 70-year period ranges from 90 trillion to 238 trillion USD_2023_. This is equivalent to between 215 and 570 USD_2023_ per ton of CO_2_ sequestered. Expected costs could be lower than these estimates as we took the conservative approach of not fully including cost savings associated with energy production from OTEC.

### Technological feasibility

The current experimental efficiencies of OTEC systems are relatively low compared to theoretical estimates likely due to the latter not accounting for the efficiencies of external components such the generator, mechanical transmission, and inverter^40^. Efforts are currently underway to improve system efficiency, primarily through turbine design which is the most influential component^40^. Non-seawater working fluids are known to increase efficiency, especially in the case of refrigerant grade R717 ammonia which is considered to be the most effective working fluid, producing up to a six-times greater power output compared to alternatives^40^. Additionally, the use of solar energy to enhance the heating of the working fluid could increase net power generated by 20–25%^41,42^.

Onshore, offshore, and floating OTEC facilities offer distinct infrastructure options for implementation. Floating facilities have the potential to be relocated depending on the available ocean vertical temperature gradient. OTEC plants could also repurpose decommissioned marine oil and gas platforms^43,44^. This oil and gas industry infrastructure has undergone decades of improvement and is known to be durable under harsh marine conditions. The use of such existing infrastructure might allow for an acceleration of the implementation of OTEC while creating a transitional pathway from the reliance on fossil fuels^44^.

### Environmental feasibility

Exploring the potential environmental consequences of widespread marine OTEC implementation is the subject of several recent studies. Modelling efforts have found ocean surface cooling in regions of OTEC implementation^9,26,45^, ocean surface warming at high latitudes^9,26,45^, and heating in the ocean interior^26,45^ due to OTEC-induced vertical mixing. Enhanced OTEC-induced vertical mixing also leads to an increased meridional gradient of depth-integrated steric height along the western boundary of the North Atlantic^46^, thereby reinforcing or even increasing the strength of the Atlantic Meridional Overturning Circulation (AMOC; 9, 26, *45*). This might be viewed as a positive environmental effect of OTEC deployment as it counteracts AMOC weakening associated with increased atmospheric greenhouse gas loading. On the other hand, the optimal location for OTEC deployment is within the warm pool of the western equatorial Pacific. Net surface cooling in this area would almost certainly affect El Niño and monsoonal systems which future research might explore.

Near-surface discharge associated with OTEC also transports large volumes of deep-nutrient rich seawater into the photic zone^45,47,48^. Artificial upwelling in nutrient depleted waters has been found to alter the assemblage of phytoplankton communities, resulting in a greater percentage of micro-phytoplankton compared to pico-phytoplankton, while primary production rates remained low^49^. Nevertheless, a mitigative solution could involve increasing the density of the discharge plume so that neutrally buoyant settling of nutrient rich waters occurs well below the photic zone thereby preventing interference with natural primary productivity levels or shifts in the composition of phytoplankton communities^44,48,49^.

Other potential biological effects associated with OTEC implementation include entrainment or impingement of marine organisms in water intake pipes^47,48^, disruption of seabed communities^47^, and changes to marine organism foraging patterns as offshore and floating OTEC infrastructure might act as fish aggregating devices that attract marine fauna potentially creating artificial reef communities^47,48,50,51^. Deep ocean organisms are highly susceptible to temperature, pH, and salinity changes^47^. Therefore, it is important to conduct field assessments at potential OTEC facility sites to establish baselines for comparison with induced changes and improve spatial planning of cumulative environmental effects^47,48^.

## Concluding remarks

As identified in ref.^1^, the goal of the Paris Accord to limit global warming to well below 2 °C above pre-industrial levels while pursuing efforts to limit the temperature increase to 1.5 °C ultimately requires the introduction and scale-up of negative emission technology. Earth has already warmed by ~ 1.1–1.2 °C since preindustrial times and if worldwide fossil fuel combustion was immediately eliminated, the direct and indirect net cooling effect of atmospheric aerosol loading would rapidly dissipate through gravitational settling and precipitation scavenging of these aerosols. As such, the source of the ~ 0.5 °C aerosol cooling realized since the preindustrial era would be eliminated, thereby taking the Earth rapidly to ~ 1.6–1.7 °C warming. The Earth would warm further as we equilibrate to the present 523 ppm CO_2_e^52^ greenhouse gas loading in the atmosphere (only 417 ppm of which is associated with CO_2_), and that is not including the committed warming from the permafrost carbon feedback that would add another 0.1 to 0.2 °C this century^53,54^.

The need to reach global net zero CO_2_ emissions this century to keep warming to below 2 °C above preindustrial times has long been recognized. For example ref.^55^, concluded that “…if a 2.0°C warming is to be avoided, direct CO_2_ capture from the air, together with subsequent sequestration, would eventually have to be introduced in addition to sustained 90% global carbon emissions reductions by 2050.” The recognition that future warming responds linearly to cumulative carbon dioxide emissions has also allowed for the calculation of probabilistic estimates of the remaining allowable carbon budget before exceeding either the 1.5–2 °C threshold^1,56–58^. Given socioeconomic inertia in the built environment, and the previous discussion, the 1.5 °C threshold will almost certainly be passed within the next few decades and even the 2 °C threshold cannot be avoided without an immediate decarbonization of energy systems and widespread deployment of negative emissions technology.

Finally, despite uncertainties in the size of the geological reservoirs available for deep ocean CO_2_ storage, our results are encouraging. The combination of OTEC and DACCS could serve as an important component of any global climate change mitigation strategy. While nature-based solutions have received a lot of attention of late (forestry, land management, blue carbon etc.), such solutions are incapable of recapturing the fossil carbon that took tens of millions of years to be created (and only a few decades to combust). Cumulative anthropogenic fossil carbon dioxide emissions from 1750 to 2021 have been 1738 GtCO_2_, while deforestation and land use changes have contributed another 744 GtCO_2_^59^. Nature-based solutions are certainly capable of addressing historical cumulative land-use carbon dioxide emissions, but they are insufficient to respond to the growing atmospheric loading of fossil carbon^60^. Given the urgency of implementing negative emission solutions, if we wish to keep global warming to a reasonable level, it may be time to consider the installation of a pilot OTEC/DACCS system in the western equatorial Indian or Atlantic Oceans. Regardless of whether OTEC/DACCS emerges as a viable global mitigation strategy, the scale of the geological sequestration required to mitigate past and future emissions suggests we are a very long way from reaching mid-century net zero emissions.

## Data Availability

The datasets used and/or analysed during the current study are available from the corresponding author upon request.

## References

[1] IPCC. : Global Warming of 1.5°C. An IPCC Special Report on the impacts of global warming of 1.5°C above pre-industrial levels and related global greenhouse gas emission pathways, in the context of strengthening the global response to the threat of climate change, sustainable development, and efforts to eradicate poverty (V. Masson-Delmotte, P. Zhai, H.-O. Pörtner, D. Roberts, J. Skea, P.R. Shukla, A. Pirani, W. Moufouma-Okia, C. Péan, R. Pidcock, S. Connors, J.B.R. Matthews, Y. Chen, X. Zhou, M.I. Gomis, E. Lonnoy, T. Maycock, M. Tignor, T. Waterfield, Eds.) Cambridge University Press, Cambridge, UK and New York, NY, USA, 616. 10.1017/9781009157940 (2018).

[2] National Academies of Sciences, Engineering, and Medicine. *Negative Emissions Technologies and Reliable Sequestration: A Research Agenda* (National Academies, 2019). 10.17226/25259 31120708

[3] Teng, Y. & Zhang, D. Long-term viability of carbon sequestration in deep-sea sediments. *Sci. Adv.* **4**, eaao6588. 10.1126/sciadv.aao6588 (2018). 29978037 10.1126/sciadv.aao6588 PMC6031374

[4] Celia, M. A., Bachu, S., Nordbotten, J. M. & Bandilla, K. W. Status of CO_2_ storage in deep saline aquifers with emphasis on modelling approaches and practical simulations. *Water Resour. Res.* **51**, 6846–6892. 10.1002/2015WR017609 (2015).

[5] Carbon Engineering Ltd. *Direct Air Capture and Storage of CO*_*2*_. accessed June 2, (2023). https://carbonengineering.com/direct-air-capture-and-storage/

[6] Rau, G. H. & Baird, J. R. Negative-CO_2_-emissions ocean thermal energy conversion. *Renew. Sustain. Energy Rev.* **95**, 265–272. 10.1016/j.rser.2018.07.027 (2018).

[7] D’Arsonval Utilisation Des forces naturelles, avenir de l’électricité. *La. Revue Scientifique de la. France et de l’étranger*. **17**, 370–372 (1881).

[8] GESAMP & /UNDP/ISA Joint Group of Experts on the Scientific Aspects of Marine Environmental Protection Rep. Stud. GESAMP No. 98, 144 pp., High level review of a wide range of proposed marine geoengineering techniques (Boyd, P.W. and Vivian, C.M.G., eds; IMO/FAO/UNESCO-IOC/UNIDO/WMO/IAEA/UN/UN Environment (2019). http://www.gesamp.org/publications/high-level-review-of-a-wide-range-of-proposed-marine-geoengineering-techniques

[9] Rajagopalan, K. & Nihous, C. G. Estimates of global ocean thermal energy conversion (OTEC) resources using an ocean general circulation model. *Renew. Energy*. **50**, 532–540. 10.1016/j.renene.2012.07.014 (2013).

[10] Kempener, R. & Neumann, F. Ocean thermal energy conversion technology brief (IRENA Ocean Energy Tech. Brief 1, (2014). https://www.irena.org/-/media/Files/IRENA/Agency/Publication/2014/Ocean_Thermal_Energy_V4_web.pdf

[11] Vega, L. A. Ocean thermal energy conversion in Encyclopedia of Sustainability Science and TechnologySpringer, pp.7296–7328. (2012). https://link.springer.com/referenceworkentry/10.1007/978-1-4419-0851-3_695

[12] Muralidharan, S. *Assessment of Ocean Thermal Energy Conversion* ( Massachusetts Institute of Technology, 2012). https://dspace.mit.edu/handle/1721.1/76927

[13] IEA. Projected Costs of Generating Electricity 2020 Edition (International Energy Agency, OECD, Paris, France). https://www.iea.org/reports/projected-costs-of-generating-electricity-2020

[14] Johnson, F. A. Closed-cycle ocean thermal energy conversion in Ocean Energy Recovery — The State of the Art, (ed Seymour, R. J.) (ASCE, New York, 70–96. (1992).

[15] Avery, W. H. & Wu, C. *Renewable Energy from the Ocean: A Guide to OTEC* (Oxford University Press, 1994). 10.1093/oso/9780195071993.001.0001

[16] Nihous, G. C. A preliminary investigation of the effect of ocean thermal energy conversion (OTEC) effluent discharge options on global OTEC resources. *J. Mar. Sci. Eng.* **6** (1), 25. 10.3390/jmse6010025 (2018).

[17] Nihous, G. C. A preliminary assessment of ocean thermal energy conversion resources. *J. Energy Res. Technol.* **129**, 10–17. 10.1115/1.2424965 (2006).

[18] Hibler, W. D. A dynamic thermodynamic sea ice model. *J. Phys. Oceanogr.* **9**, 815–846. (1979).

[19] Hunke, E. C. & Dukowicz, J. K. An elastic–viscous–plastic model for sea ice dynamics. *J. Phys. Oceanogr.* **27**, 1849–1867. (1997).

[20] Bitz, C. M. & Lipscomb, W. H. An energy-conserving thermodynamic model of sea ice. *J. Geophys. Res. Oceans*. **104**, 15669–15677. 10.1029/1999JC900100 (1999).

[21] Pacanowski, R. C. MOM 2 version 2.0 beta documentation user’s guide and reference manualGFDL Ocean Tech. Rep. 3.2, NOAA GFDL, (1996). https://www.gfdl.noaa.gov/wp-content/uploads/2016/10/manual2.2.pdf

[22] Weaver, A. J. et al. The UVic Earth System Climate Model: Model description, climatology, and applications to past, present and future climates. *Atmos. Ocean*. **39**, 361–428. 10.1080/07055900.2001.9649686 (2001).

[23] Moss, R. H. et al. The next generation of scenarios for climate change research and assessment. *Nature* **463**, 747–756 (2010). 20148028 10.1038/nature08823

[24] Zickfeld, K. et al. Long-term climate change commitment and reversibility: an EMIC intercomparison. *J. Clim.* **16**, 5782–5809. 10.1175/JCLI-D-12-00584.1 (2013).

[25] Nihous, G. C. An order-of-magnitude estimate of ocean thermal energy conversion resources. *J. Energy Res. Technol.* **127**, 328–333. 10.1115/1.1949624 (2005).

[26] Jia, Y., Nihous, G. C. & Rajagopalan, K. An evaluation of the large-scale implementation of ocean thermal energy conversion (OTEC) using an ocean general circulation model with low-complexity atmospheric feedback effects. *J. Mar. Sci. Eng.* **6**, 12. 10.3390/jmse6010012 (2018).

[27] Keith, D. W., Holmes, G., St. Angelo, D. & Heidel, K. A process for capturing CO_2_ from the atmosphere. *Joule* **2**, 1573–1594. 10.1016/j.joule.2018.05.006 (2018).

[28] IEA, *Direct Air Capture 2022: A key Technology for Net Zero*, IEA, Paris (2022). https://www.iea.org/reports/direct-air-capture-2022

[29] Benson, S. et al. Chapter 5. Underground geological storage in IPCC Special Report on Carbon Dioxide Capture and Storage, (eds Metz, B., Davidson, O., de Coninck, H., Loos, M. & Meyer, L.) (Cambridge University Press, 195–276. https://www.ipcc.ch/site/assets/uploads/2018/03/srccs_chapter5-1.pdf. (2005).

[30] de Coninck, H. et al. in *Chapter 4. Strengthening and Implementing the Global Response in Global Warming of 1.5°C*. 313–444 (eds Masson-Delmotte, V., Zhai, P., Pörtner, H. O., Roberts, D., Skea, J., Shukla, P. R., Pirani, A., Moufouma-Okia, W., Péan, C., Pidcock, R., Connors, S., Matthews, J. B. R., Chen, Y., Zhou, X., Gomis, M. I., Lonnoy, E., Maycock, T., Tignor, M. & Waterfield, T.) (Cambridge University Press, 2018). 10.1017/9781009157940.006

[31] House, K. Z. et al. Economic and energetic analysis of capturing CO_2_ from Ambient Air. *Proceedings of the National Academy of Sciences* 108, 20428–20433 (2011). 10.1073/pnas.1012253108 10.1073/pnas.1012253108 PMC3251141 22143760

[32] Lackner, K. S. The promise of negative emissions. *Science* **354**, 714–714. 10.1126/science.aal2432 (2016). 27846596 10.1126/science.aal2432

[33] Lackner, K. S., Brennan, S., Matter, J. M. & van der Zwaan, B. The urgency of the development of CO_2_ capture from ambient air. *Proceedings of the National Academy of Sciences*, 109, 13156–13162. 10.1073/pnas.1108765109 10.1073/pnas.1108765109 PMC3421162 22843674

[34] Socolow, R. et al. Direct air capture of CO_2_ with chemicals: a technology assessment for the APS panel on public affairs. *(American Phys. Soc.*, (2011). https://www.aps.org/policy/reports/assessments/upload/dac2011.pdf

[35] Service, R. F. Cost plunges for capturing carbon dioxide from the air. *Science*10.1126/science.aau4107 (2018).30573611

[36] Stevens, S. H., Kuuskra, V. A., Gale, J. & Beecy, D. CO_2_ injection and sequestration in depleted oil and gas fields and deep coal seams: worldwide potential and costs. *Environ. Geosci.* **8**, 200–209. 10.1046/j.1526-0984.2001.008003200.x (2001).

[37] Nunez-Lopez, V. & Moskal, E. Potential of CO_2_-EOR for near-term decarbonizaton. *Front. Clim.* **1**, 5. 10.3389/fclim.2019.00005 (2019).

[38] Middleton, R. S. A new optimization approach to energy network modeling: anthropogenic CO_2_ capture coupled with enhanced oil recovery. *Int. J. Energy Res.* **37**10.1002/er.2993 (2012).

[39] Caldeira, K. et al. Chapter 6 Ocean storage in IPCC Special Report on Carbon Dioxide Capture and Storage, (eds Metz, B., Davidson, O., de Coninck, H., Loos, M. & Meyer, L.) (Cambridge University Press, 277–318. https://www.ipcc.ch/site/assets/uploads/2018/03/srccs_chapter6-1.pdf. (2018).

[40] Chen, F. et al. Theoretical and experimental research on the thermal performance of ocean thermal energy conversion system using the rankine cycle mode. *Energy* **183**, 497–503. 10.1016/j.energy.2019.04.008 (2019).

[41] Yamada, N., Hoshi, A. & Ikegami, Y. Performance simulation of solar-boosted ocean thermal energy conversion plant. *Renew. Energy*. **34** (7), 1752–1758. 10.1016/j.renene.2008.12.028 (2009).

[42] Aydin, H., Lee, H. S., Kim, H. J., Shin, S. K. & Park, K. Off-design performance analysis of a closed-cycle ocean thermal energy conversion system with solar thermal preheating and superheating. *Renew. Energy*. **72**, 154–163. 10.1016/j.renene.2014.07.001 (2014).

[43] Adiputra, R., Utsunomiya, T., Koto, J., Yasunaga, T. & Ikegami, Y. Preliminary design of a 100 MW-net ocean thermal energy conversion (OTEC) power plant study case: Mentawai island, Indonesia. *J. Mar. Sci. Technol.* **25** (1), 48–68. 10.1007/s00773-019-00630-7 (2020).

[44] IEA Technology Programme for Ocean Energy Systems, White Paper on Ocean Thermal Energy Conversion (OTEC). The Executive Committee of the IEA Ocean Energy Systems (OES). (2021). www.ocean-energy-systems.org

[45] Nickoloff, A. G., Olim, S. T., Eby, M. & Weaver, A. J. Environmental Impacts from Widespread Implementation of Ocean Thermal Energy Conversion. *Climatic Change*, in revision. 10.1007/s10584-025-03944-1 PMC12064605 40352813

[46] Hughes, T. H. C. & Weaver, A. J. Multiple equilibria of an asymmetric two-basin ocean model. *J. Phys. Oceanogr.* **24** (3), 619–637. (1994).

[47] Hammar, L. et al. Introducing ocean energy industries to a busy marine environment. *Renew. Sustain. Energy Rev.* **74**, 178–185. 10.1016/j.rser.2017.01.092 (2017).

[48] Comfort, C. M. & Vega, L. Environmental assessment for ocean thermal energy conversion in Hawaii: available data and a protocol for baseline monitoring. *OCEANS’11 MTS/IEEE KONA*. 1–8. 10.23919/OCEANS.2011.6107210 (2011).

[49] Giraud, M. et al. Potential effects of deep seawater discharge by an Ocean Thermal Energy Conversion plant on the marine microorganisms in oligotrophic waters. *Sci. Total Environ.* **693**, 133491. 10.1016/j.scitotenv.2019.07.297 (2019). 31362231 10.1016/j.scitotenv.2019.07.297

[50] Coastal Response Research Center. *Ocean Thermal Energy Conversion: Assessing Potential Physical, Chemical and Biological Impacts and Risks* (University of New Hampshire, 2010).

[51] Russell, D. J. F. et al. Marine mammals trace anthropogenic structures at sea. *Curr. Biol.* **24** (14), R638–R639. 10.1016/j.cub.2014.06.033 (2014). 25050956 10.1016/j.cub.2014.06.033

[52] NOAA, The NOAA annual greenhouse gas index. (2022). https://gml.noaa.gov/aggi/aggi.html (accessed May 9, 2023).

[53] MacDougall, A. H., Avis, C. A. & Weaver, A. J. Significant contribution to warming from the permafrost carbon feedback. *Nat. Geosci.* **5**, 719–721. 10.1038/ngeo1573 (2012).

[54] MacDougall, A. H., Eby, M. & Weaver, A. J. If anthropogenic carbon emissions cease, will atmospheric CO2 concentration continue to increase? *J. Clim.* **26**, 9563–9576. 10.1175/JCLI-D-12-00751.1 (2013).

[55] Weaver, A. J., Zickfeld, K., Montenegro, A. & Eby, M. Long term climate implications of 2050 emission reduction targets. *Geophys. Res. Lett.* **34**, L19703. 10.1029/2007GL031018 (2007).

[56] Weaver, A. J. *Keeping our Cool: Canada in a Warming World*323 (Viking Canada, Toronto, 2008).

[57] Zickfeld, K., Eby, M., D. Matthews, H. & J. Weaver, A. Setting cumulative emissions targets to reduce the risk of dangerous climate change. *Proc. Natl. Acad. Sci.* **106**, 16129–16134. 10.1073/pnas.080580010 (2009). 19706489 10.1073/pnas.0805800106 PMC2752604

[58] Allen, M. R. et al. Warming caused by cumulative carbon emissions towards the trillionth tonne. *Nature* **458**, 1163–1166. 10.1038/nature08019 (2009). 19407800 10.1038/nature08019

[59] Friedlingstein, P. et al. Global carbon budget 2022. *Earth Syst. Sci. Data*. **14**, 4811–4900. 10.5194/essd-14-4811-2022 (2022).

[60] Allen, M. R. et al. Geological net zero and the need for disaggregated accounting for carbon sinks. *Nature*10.1038/s41586-024-08326-8 (2024). 39557072 10.1038/s41586-024-08326-8

